# The Prevalence of Familial Hypercholesterolemia (FH) in Chinese Patients With Acute Myocardial Infarction (AMI): Data From Chinese Acute Myocardial Infarction (CAMI) Registry

**DOI:** 10.3389/fcvm.2020.00113

**Published:** 2020-07-16

**Authors:** Hui-Wei Shi, Jin-Gang Yang, Yang Wang, Wei Li, Yuan-Lin Guo, Ying Gao, Yi-Da Tang, Jian-Jun Li, Na-Qiong Wu, Yue-Jin Yang

**Affiliations:** ^1^Endocrinology and Cardiometabolic Center, State Key Laboratory of Cardiovascular Disease, National Center for Cardiovascular Diseases, Fuwai Hospital, Chinese Academy of Medical Sciences and Peking Union Medical College, Beijing, China; ^2^Medical Research and Biometrics Center, National Center for Cardiovascular Diseases, Beijing, China

**Keywords:** acute myocardial infarction, heterozygous familial hypercholesterolemia, prevalence, Chinese, clinical manifestations

## Abstract

**Background:** The prevalence of familial hypercholesterolemia (FH) in the patients with acute myocardial infarction (AMI) in China is unclear.

**Materials and Methods:** In China Acute Myocardial Infarction (CAMI) Registry, 13,002 patients with age 18–80 were consecutively enrolled with first-onset AMI who were naïve to statin before admission from January 1st, 2013 to October 31st, 2014. According to Dutch Lipid Clinical Network Criteria (DLCNC), the patients were divided to heterozygous familial hypercholesterolemia (HeFH) (definite/probable HeFH, possible HeFH) or non-HeFH group.

**Results:** The number of the patients in the three groups was as following, 62 in definite/probable HeFH group, 484 in possible HeFH group, 12,456 in non-HeFH group. The prevalence of HeFH is 4.2% (including 0.47% of definite/probable HeFH, 3.73% of possible FH). The average age of onset of first-time AMI was 54 ± 12, 56 ± 12, 63 ± 12 years old (*p* < 0.0001) in definite/probable HeFH group, possible HeFH group and non-HeFH group, respectively. The percentage of Killip III or above (8.1 vs. 4.3 vs. 6.3%, *p* = 0.1629), cardiac arrest (1.6 vs. 0.6 vs. 0.9%, *p* = 0.6990), and TIMI 0–2 grade after primary percutaneous cardiac intervention (PCI) (0 vs. 6.8 vs. 4.3%, *p* = 0.5866) was not significantly different in definite/probable HeFH group, possible HeFH group and non-HeFH group, respectively.

**Conclusions:** The prevalence of HeFH in Chinese patients with AMI is 4.2%. The patients were significantly younger in HeFH group, when compared with those with non-HeFH. However, no significant differences were found in the severity of clinical manifestations in both HeFH and non-HeFH group.

## Introduction

Characteristic of elevated blood cholesterol and increased CHD susceptibility, familial hypercholesterolemia (FH) presents a dominant genetic disorder which leads to dysregulated lipid metabolism. The acknowledged causes of FH include defects in the low-density lipoprotein (LDL)-receptor (LDLR), apolipoprotein B (APOB), proprotein convertase subtilisin/kexin type 9 (PCSK9), and LDL receptor adaptor protein 1 (LDLRAP1) (1). Heterozygous FH (HeFH) is a common type of FH, which is characterized with extremely raised LDL cholesterol levels and the occurrence of premature coronary heart disease (CHD). Unfortunately, FH remains largely underdiagnosed and undertreated in the general population, and many patients with FH are diagnosed only at the time of their first coronary event (2). After all, diagnosis of FH is primarily dependent on clinical manifestation with subsequent affirmation by genetic testing when necessary and available (1).

Specifically, FH is a frequent disorder among patients with ACS. In Europe, up to 8% of adults hospitalized for acute coronary syndromes (ACS) have been shown to have clinical criteria compatible with FH (3, 4), which is >10 times greater than the prevalence of FH in the general population. Previous data have suggested that among the subjects with recognized cardiovascular diseases, the prevalence of FH deserves attention. Studies conducted by our group had pointed out that the prevalence of probable and definite FH was 5.8% in Chinese patients with coronary artery disease (CAD) (5) and 3.9% in patients with myocardial infarction (MI) (6). However, even if FH is frequent among patients with premature CHD, there is still no established screening strategy to decrease the number of missed diagnoses, leaving patients with FH to a higher risk in CHD (7). Early screening is a significant measure to improve clinical prognosis and to reduce the incidence of ASCVD in patients with FH. According to experts (8), patients who meet any criteria listed below should be considered into systemic screening: the occurrence of premature ASCVD (<55 years old for male and <65 years old for female), the plasma level of LDL-C ≥ 3.8 mmol/L (146.7 mg/dl) for adults and ≥2.9 mmol/L (112.7 mg/dl) for children, physical examination of xanthomas and corneal arcus for patients below 45 years old, their first-degree relatives presenting any of the three cases mentioned above. What the screening procedure involves are inquiry of family history and personal clinical history as well as physical examination, with plasma LDL-C concentration detection also included. The guidelines also suggest that when patients are found to be with FH, cascade screening targeted to their first-degree relatives should be performed.

Cascade screening (CS) serves as an effective method for FH screening and a targeted strategy to detect FH mutation and identify new FH index cases traced from family members, and it is directed at specific patient groups who are likely to show a high prevalence of FH such as those with post-acute coronary syndrome (9). Moreover, when combined with clinical criteria, Next Generation Sequencing (NGS), which undertakes parallel sequencing relatively, has been proved to be of extremely high levels of specificity and sensitivity in identifying FH (10). However, limited by inadequate budgets and counseling, unaffordable genetic testing may be hard to be carried out in clinical practice. Hence, the Dutch Lipid Clinic Network (DLCN) criteria in terms of family and personal history of premature CHD as well as physical examination and plasma LDL-C concentration is considered as the most widely accepted approach for clinical diagnosis. In recent studies, in Italian patients with established CAD, it indicated the percentage of DLCN score over 6 was 3.7% (11). Genetic analysis is recommended if the DLCN score is >5.

However, FH remains widely unidentified and undertreated worldwide (2). If not recognized and treated at an early age, patients with FH will have a dramatically increased lifetime risk of CHD, which is 20-fold higher than that in general population (12). For adults with or without ASCVD, the target concentration of plasma LDL-C is <1.8 mmol/L (70 mg/dl) (13) and <2.6 mmol/L (8) (100 mg/dl) respectively; While for children with FH, the target value is <3.4 mmol/L (130 mg/dl). And the level of LDL-C is recommended to reach a reduction of more than 50% when the above target values are difficult to reach (8). Clinical trials support the effectiveness and safety of statins in decreasing CHD events in both primary and secondary settings (14) and intensive lipid lowering treatment for FH patients decreases the risk of developing CHD and onset of AMI (15). Management of cardiovascular risk factors and available lipid-lowering therapy are proved to be contributing elements in controlling the development of FH (16). For patients whose lipid targets on statin monotherapy are not achieved or who are of statins intolerance, small intestine cholesterol absorption blocking agent Ezatimbe is accepted to use in combination with statin application. Other available agents proven to be efficacious are referred to mipomersen and lomitapide, with the former acts as an inhibitor of apolipoprotein B-100 synthesis, the latter as an inhibitor of microsomal triglyceride transfer protein (1). In dealing with patients with HoFH, high-intensity statin therapy, and LDL apheresis are recommended (17).

Moreover, prognosis data are lacking in patients with FH in the setting of acute myocardial infarction (AMI), particularly in the era of widespread statin use. Recent research has suggested Familial Hypercholesterolaemia Case Ascertainment Tool (FAMCAT) to be a most sensitive prognostic model (18), whose criteria are based on family history details such as MI, FH, and elevated cholesterol levels. However, very few data document the likelihood of cardiovascular event recurrence in patients diagnosed as AMI with FH compared with those with no FH. This study aimed to investigate the prevalence of clinical HeFH among Chinese patients with first onset AMI.

## Subjects and Methods

### Subjects

**Figure 1:**
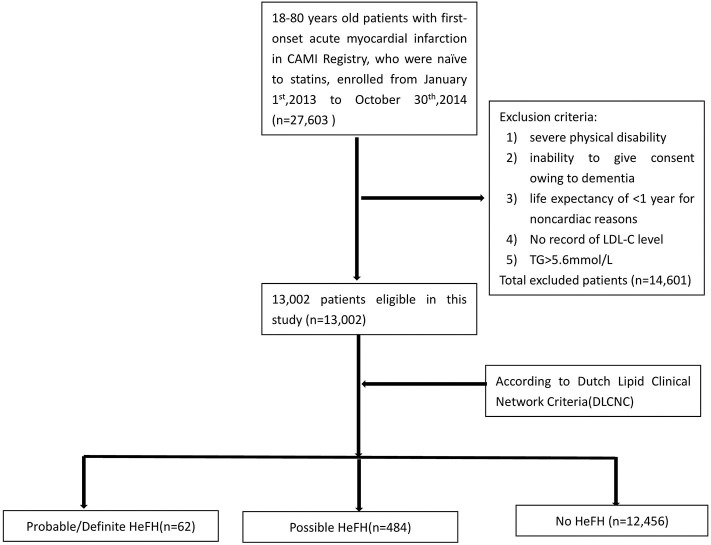
Flowchart of the enrolled patients.

In China Acute Myocardial Infarction (CAMI) Registry, 27,603 patients with age 18–80 were consecutively enrolled with first-onset AMI who were naïve to statins before admission from January 1st, 2013 to October 31st, 2014. Inclusion criteria were a main diagnosis of ST-elevation–elevation myocardial infarction (STEMI) for patients presenting after pain onset, non-ST-segment–elevation myocardial infarction (NSTEMI). Exclusion criteria included severe physical disability, inability to give consent owing to dementia, and life expectancy of <1 year for non-cardiac reasons, TG level ≥ 500 mg/dL and LDL-C records unavailable. Totally, 14,601 patients were excluded. Finally, 13,002 patients were analyzed in this study (Figure 1). According to Dutch Lipid Clinical Network (DLCN) criteria, the patients were divided into HeFH group (including probable/definite HeFH group and possible HeFH group) and non-HeFH group.

### Diagnosis of HeFH

Clinical HeFH was diagnosed using the DLCN criteria including personal and family history of premature atherosclerosis, physical examination of xanthomas, and corneal arcus, and elevated LDL-C levels. Based on the DLCN criteria, numerical scores were assigned as follows: (1) family history of a first-degree relative with known premature CHD or vascular disease (<55 years for men, <60 years for women) (1 point) and/or a first-degree relative with known hypercholesterolemia (1 point) or offspring(s) with known hypercholesterolemia (2 points). (2) personal history of premature CHD (age as above, 2 points) or cerebral/peripheral vascular disease (ages as above, 1 point) or xanthomas (6 points); untreated LDL-C > 8.5 mmol/L (8 points), 6.5–8.4 mmol/L (5 points), 5.0–6.4 mmol/L (3 points), or 4.0–4.9 mmol/L (1 point); (3) Xanthomas signs, corneal arcus, and genetic diagnosis were not available, and these missing information were counted as zero. Finally, a diagnosis of definite FH was considered if the total score was >8 points, probable if the score was 6–8 points, possible if the score was 3–5 points, and unlikely if the score was <3 points. Xanthomas signs and genetic tests of FH were counted as zero in all definitions because such information was missing for too many patients according to the method which was introduced in the previous study (19).

### Covariables

Total cholesterol (TC), high-density lipoprotein cholesterol (HDL-C), and triglycerides (TG) levels were measured in the first blood draw at the emergency department within 24 h of admission and immediately processed locally with standardized and certified dose methods. LDL cholesterol was calculated with the Friedewald formula when TG levels were <500 mg/dL. LDL cholesterol was considered missing for patients with triglyceride levels of ≥500 mg/dL. Personal history of premature CHD was considered positive when men were <55 years old and women were <60 years old at the time of first AMI. Family history of premature CHD was based on patient reports of a coronary event in a brother or father <55 years old or a mother or sister <60 years old. Hypertension was defined as a systolic blood pressure ≥140 mm Hg or diastolic blood pressure ≥90 mm Hg or use of blood pressure-lowering drugs. Blood pressure-lowering drugs included all medications in the classes of angiotensin-converting enzyme inhibitors, angiotensin II receptor blockers, β-blockers, calcium channel blockers, diuretics, and nitrates. Smoking status was categorized into current, former, and never smokers. Former smokers were those who smoked at least 1 cigarette a day during at least 1 year and were non-smokers for >1 month before inclusion. Diabetes mellitus was either self-reported or diagnosed by the use of antihyperglycemic medication or a hemoglobin A1c of ≥6.5% at admission.

### Statistical Analysis

At the time of hospitalization for AMI, we categorized patients according to the presence of HeFH according to DLCN criteria and reported clinical characteristics in each group, as well as in patients not selected by the definition of HeFH. All hypothesis tests were two-sided, and the significance level was set at 5%. Statistical analyses were performed with STATA 14 (STATA Corp, College Station, TX).

### Ethics Statement

Our study complied with the Declaration of Helsinki and was approved by the hospital's ethical review board (FuWai Hospital, Beijing, China). Informed written consent was obtained from all patients enrolled in this study.

## Results

**Figure 2:**
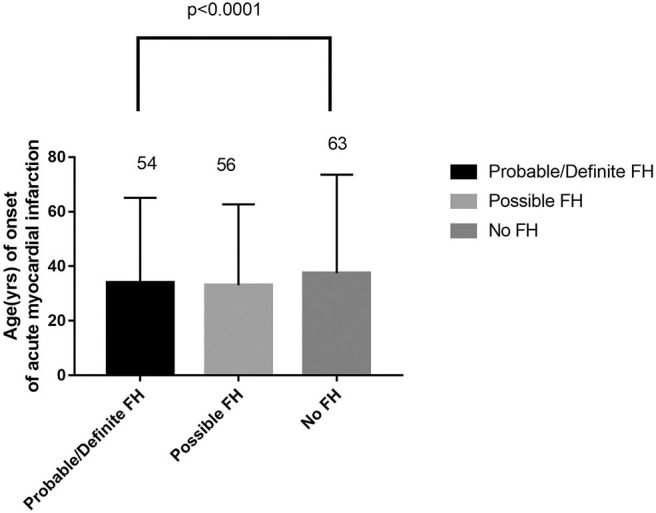
Comparison about age of onset of acute myocardial infarction in probable/definite HeFH, possible HeFH, and non-HeFH.

**Figure 3:**
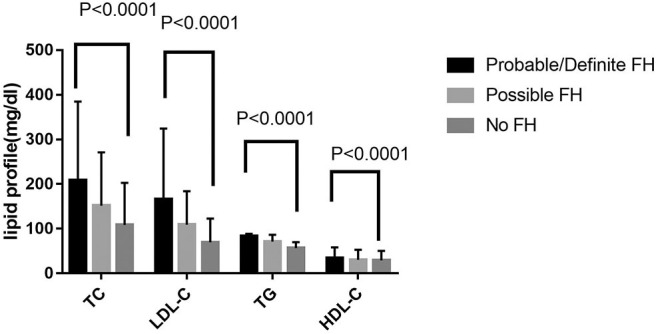
Comparison about lipid profiles among probable/definite HeFH, possible HeFH, and non-HeFH groups.

**Figure 4:**
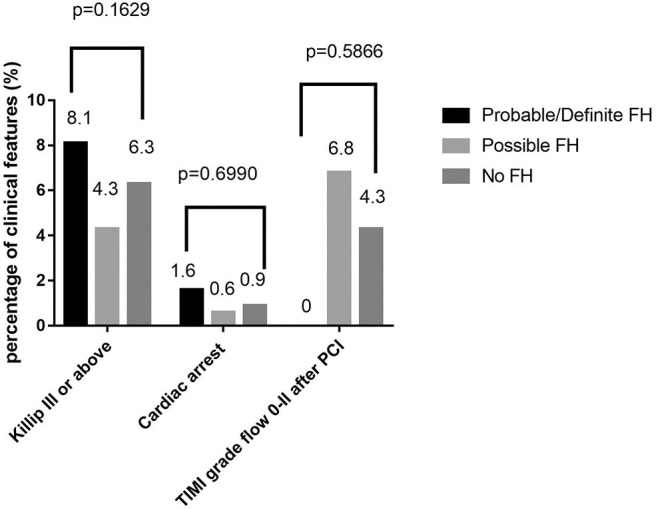
Comparison about severe clinical manifestations among probable/definite HeFH, possible HeFH, and non-HeFH groups.

Among 13,002 patients hospitalized with first-onset AMI, 484 (3.73%) were identified with possible FH and a further 62 (0.47%) with probable/definite FH with the Dutch Lipid Clinic definition. The prevalence of HeFH is 4.2% (including 0.47% of definite/probable HeFH, 3.73% of possible FH). Overall, 12,456(95.8%) of the total number of patients with AMI didn't reach the criteria for HeFH. Clinical characteristics of patients with and without HeFH were reported in Table 1. The average age of onset of first-time AMI was 54 ± 12, 56 ± 12, and 63 ± 12 years old (*p* < 0.0001) in definite/probable HeFH group, possible HeFH group, and non-HeFH group, respectively. Compared with the patients without HeFH, patients with HeFH were 7–9 years younger (Figure 2). The percentage of hypertension was not significantly different in definite/probable HeFH group, possible HeFH compared with non-HeFH group (48.4 vs. 48.6 vs. 48.0%, *p* = 0.7958). The percentage of DM was not significantly different in definite/probable HeFH group, possible HeFH compared with non-HeFH group (13.1 vs. 20.3 vs. 17.8%, *p* = 0.2933). The levels of eGFR were not significantly different in the three groups. The percentage of statins prescription at discharge was 94, 92, and 93%, respectively (*p* > 0.05) among the three groups (Table 1). The levels of TC were 333 ± 83 vs. 236 ± 67 vs.175 ± 42 mg/dL, *p* < 0.0001, and the levels of LDL-C was significantly higher in both definite/probable HeFH and possible HeFH group than in non-HeFH group (278 ± 54 vs.162 ± 56 vs.107 ± 32 mg/dL, *p* < 0.0001). The levels of TG and HDL-C were also higher in both definite/probable HeFH and possible HeFH group than in non-HeFH group (TG: 87 ± 79 vs. 82 ± 60 vs. 66 ± 47 mg/dL, *p* < 0.0001; HDL-C: 51 ± 17 vs. 46 ± 14 vs. 44 ± 14 mg/dL, *p* < 0.0001; Figure 3). The percentage of Killip III or above (8.1 vs. 4.3 vs. 6.3%, *p* = 0.1629), cardiac arrest (1.6 vs. 0.6 vs. 0.9%, *p* = 0.6990), and TIMI 0–2 grade after primary percutaneous cardiac intervention (PCI) (0 vs. 6.8 vs. 4.3%, *p* = 0.5866) was not significantly different in definite/probable HeFH, possible HeFH, and non-HeFHgroup, respectively (Figure 4).

**Table 1:** Baseline characteristics of all the enrolled patients in the different groups which divided by the DLCN criteria.

	**Probable/DefiniteFH (>5 Points) (*n* = 62)**	**Possible FH (3–5 Points) (*n* = 484)**	**No FH (0– <3 points) (*n* = 12,456)**	***P*-value**
Percentage (%)	0.47	3.73	95.80	
Age, year	56 ± 12	54 ± 12	63 ± 12	<0.0001
Female, *n* (%)	31 (50.0)	152 (31.4)	3,327 (26.7)	<0.0001
Body mass index, kg/m^2^	25 ± 3	25 ± 4	24 ± 10	0.3769
Current smoking	18 (29.0)	243 (50.3)	5,656 (45.6)	<0.0001
Former smoking	2 (3.2)	25 (5.2)	1,186 (9.6)	<0.0001
Elevated alcohol use	22 (35.5)	177 (36.6)	4,842 (39.0)	0.1145
Hypertension, *n* (%)	30 (48.4)	235 (48.6)	5,977 (48.0)	0.7958
DM, *n* (%)	8 (13.1)	98 (20.3)	2,219 (17.8)	0.2933
Premature CHD, *n* (%)	15 (24.2)	236 (48.9)	220 (1.8)	<0.0001
Killip III or above, *n* (%)	5 (8.1)	21 (4.3)	779 (6.3)	0.1629
Cardiac arrest, *n* (%)	1 (1.6)	3 (0.6)	109 (0.9)	0.6990
TIMI 0–2 after p PCI, *n* (%)	0 (0)	12 (6.8)	166 (4.3)	0.5866
Total cholesterol, mg/dl	333 ± 83	236 ± 67	175 ± 42	<0.0001
LDL cholesterol, mg/dl	278 ± 54	162 ± 56	107 ± 32	<0.0001
HDL cholesterol, mg/dl	51 ± 17	46 ± 14	44 ± 14	<0.0001
Triglycerides, mg/dl	87 ± 79	82 ± 60	66 ± 47	<0.0001
Statin prescription at discharge, *n* (%)	56 (90.3)	438 (92.4)	11,138 (92.8)	0.7384

## Discussion

In this multicenter registry study, we provided the information about the prevalence of HeFH (which was diagnosed according to DLCNC) in the patients with first-onset AMI in China. The prevalence rate of FH was 4.2% (depending on which clinical definition of FH was used) among patients hospitalized for first-onset AMI. It was almost 8 times higher concerning the prevalence of HeFH in general population which is estimated as 1/200 (20). It demonstrated that the diagnosis of HeFH was an important risk factor for AMI.

This is the largest investigation in China, where there was lack of detailed data before. In some studies, HeFH is a frequent disorder among patients with ACS, with prevalence rates reaching 15% (3, 4, 21, 22). Surprisingly, in our study, the prevalence of HeFH (including definite/probable HeFH and possible HeFH) was relatively low. The possible reasons were listed as following, the recording bias about the LDL-C level (2′ in DLCN criteria) or the history of premature cardiovascular disease in the first relatives (1′ in DLCN criteria), and counted as zero including xanthomas signs (6′ in DLCN criteria), corneal bow (4′ in DLCN criteria) and genetic tests (8′ in DLCN criteria) due to such informations missing in too many patients.

Although genetic testing is considered as the gold standard of FH diagnosis and there exists no internationally accepted and accurate diagnostic standard, however, in the other hand, in clinical practice, the implementation of a systematic screening strategy for FH is limited by the costs of genetic testing and counseling (23). Hence, the Dutch Lipid Clinic Network (DLCN) criteria in terms of clinical manifestation is considered as the most widely accepted approach for clinical diagnosis, exceeding the two other clinically proven diagnostic tools the Simon Broome Register (SBR) (24) criteria applied in the UK and the Make Early Diagnosis to Prevent Early Death (MEDPED) (25) criteria in the USA. Different from SBR criteria regarding the detection of DNA mutation as an identified definition of FH, DLCN also needs additional clinical criteria to reach a definite diagnosis of FH. One study carried out in Dutch lipid clinics prospectively examined the rates of coronary events in patients with FH without a record of pre-existing cardiovascular disease (26). Other studies in Dutch lipid clinics were retrospective or cross-sectional, and cardiovascular risk estimates may have been prone to confounding biases (27, 28). Because cardiovascular risk was shown to be driven by both phenotype and genotype (28, 29), expert groups proposed simplified definitions for establishing a diagnosis of FH based on clinical criteria (30–32). In our study, we tried to demonstrate how these simplified screening algorithms using cholesterol levels and family or personal history of premature CHD as diagnostic criteria were powerful and efficient tools to identify FH in patients with AMI at high risk of coronary event recurrence. Systematic screening for FH based on clinical criteria has been shown to be easy to carry out and affordable. With the added benefits that PCSK9 inhibitors are expected to bring in terms of cardiovascular events reduction, systematic screening for FH should be encouraged in patients with AMI until further evidence is available.

Importantly, we found that the average age for patients with first-onset AMI was 7–9 years younger in either definite/probable HeFH group or possible HeFH group compared with those in non-HeFH group. It suggested that the diagnosis of HeFH was possibly a strong risk factor for premature CHD. The result was in accordance with previous result reported (33).

### Limitations

There were some limitations in this study. Firstly, although in our study the baseline characteristics showed that high percentages of statin prescription at discharge among the three groups, no details of LDL-C level and further analysis about the rate of cardiovascular diseases between the groups during follow-up and no data about the percentage of LDL-C goal achievement for these patients was the shortage of our study.

Secondly, there is no denying that our results was limited by lack of molecular diagnosis. In the procedure of FH identification, genetic testing serves as a significant role of subsequent confirmation after uncertainty from clinical diagnosis, getting carried out when the DLCN score is >5. Moreover, identification of a causal molecular mutation may provide additional motivation for some patients to implement appropriate treatment.

More importantly, consistent with previous relevant studies, information regarding genetic testing of FH, and Xanthomas signs was missing for a majority of patients, thus the DLCN score about the two issues was counted as zero in all definitions, which gave rise to a tendency to a lower total score. Accordingly, in our study, we added the proportion of possible HeFH to the total prevalence of HeFH. This thus led to increased sensitivity and reduced specificity of HeFH diagnosis.

## Conclusions

The prevalence of HeFH in Chinese patients with AMI is 4.2%. The patients were significantly younger in HeFH group, when compared with those with non-HeFH. However, no significant differences were found in the severity of clinical manifestations between HeFH and non-HeFH group. In the other hand, identification of HeFH in those AMI patients could be helpful to achieve more intensive management of LDL-C to improve their long-term prognosis. Further investigations should be performed about the prevalence and prognosis of HeFH in AMI patients in China.

## Data Availability Statement

The raw data supporting the conclusions of this article will be made available by the authors, without undue reservation.

## Ethics Statement

The studies involving human participants were reviewed and approved by the hospital's ethical review board (FuWai Hospital, Beijing, China). The patients/participants provided their written informed consent to participate in this study.

## Author Contributions

N-QW and Y-JY were in charge of the design of this study. H-WS, N-QW, J-GY, Y-LG, YG, Y-DT, J-JL, and on behalf of CAMI Registry study group carried out the experiments. H-WS, N-QW, YW, and WL mainly analyzed the experiments data and results. H-WS, N-QW, and Y-JY wrote the manuscript. All authors contributed to the article and approved the submitted version.

## Conflict of Interest

The authors declare that the research was conducted in the absence of any commercial or financial relationships that could be construed as a potential conflict of interest.

## Abbreviations

FH: Familial Hypercholesterolemia
AMI: Acute Myocardial Infarction
CAMI: Chinese Acute Myocardial Infarction
DLCNC: Dutch Lipid Clinical Network Criteria
HeFH: heterozygous familial hypercholesterolemia
LDLR: low-density lipoprotein (LDL)-receptor
APOB: apolipoprotein B
PCSK9: proprotein convertase subtilisin/kexin type 9
LDLRAP1: LDL receptor adaptor protein 1
CHD: coronary heart disease
ACS: acute coronary syndromes
STEMI: ST-elevation–elevation myocardial infarction
NSTEMI: non-ST-segment-elevation myocardial infarction.
