# Corrigendum: The Prevalence of Familial Hypercholesterolemia (FH) in Chinese Patients With Acute Myocardial Infarction (AMI): Data From Chinese Acute Myocardial Infarction (CAMI) Registry

**DOI:** 10.3389/fcvm.2020.00160

**Published:** 2020-08-26

**Authors:** Hui-Wei Shi, Jin-Gang Yang, Yang Wang, Wei Li, Yuan-Lin Guo, Ying Gao, Yi-Da Tang, Jian-Jun Li, Na-Qiong Wu, Yue-Jin Yang

**Affiliations:** ^1^Endocrinology and Cardiometabolic Center, State Key Laboratory of Cardiovascular Disease, National Center for Cardiovascular Diseases, Fuwai Hospital, Chinese Academy of Medical Sciences and Peking Union Medical College, Beijing, China; ^2^Medical Research and Biometrics Center, National Center for Cardiovascular Diseases, Beijing, China

**Keywords:** acute myocardial infarction, heterozygous familial hypercholesterolemia, prevalence, Chinese, clinical manifestations

In the original article, there was a mistake in Figure 2 and Figure 4 as published. ***The content*** ***of figure 2 and figure 4 were dislabled while the legends below were in correct position*, **the two figures should be swaped for their positions, the Figure 2 captioned as **“*Comparison*** ***about age of onset of acute myocardial infarction in probable/definite HeFH, possible*** ***HeFH, and non-HeFH”***should be the first figure presented as follows; the Figure 4 captioned as **“*Comparison about severe clinical manifestations among probable/definite*** ***HeFH, possible HeFH, and non-HeFH groups.”***should be the second figure presented as follows.

The corrected presentation of Figure 2 and Figure 4 appears below.

**Figure 2:**
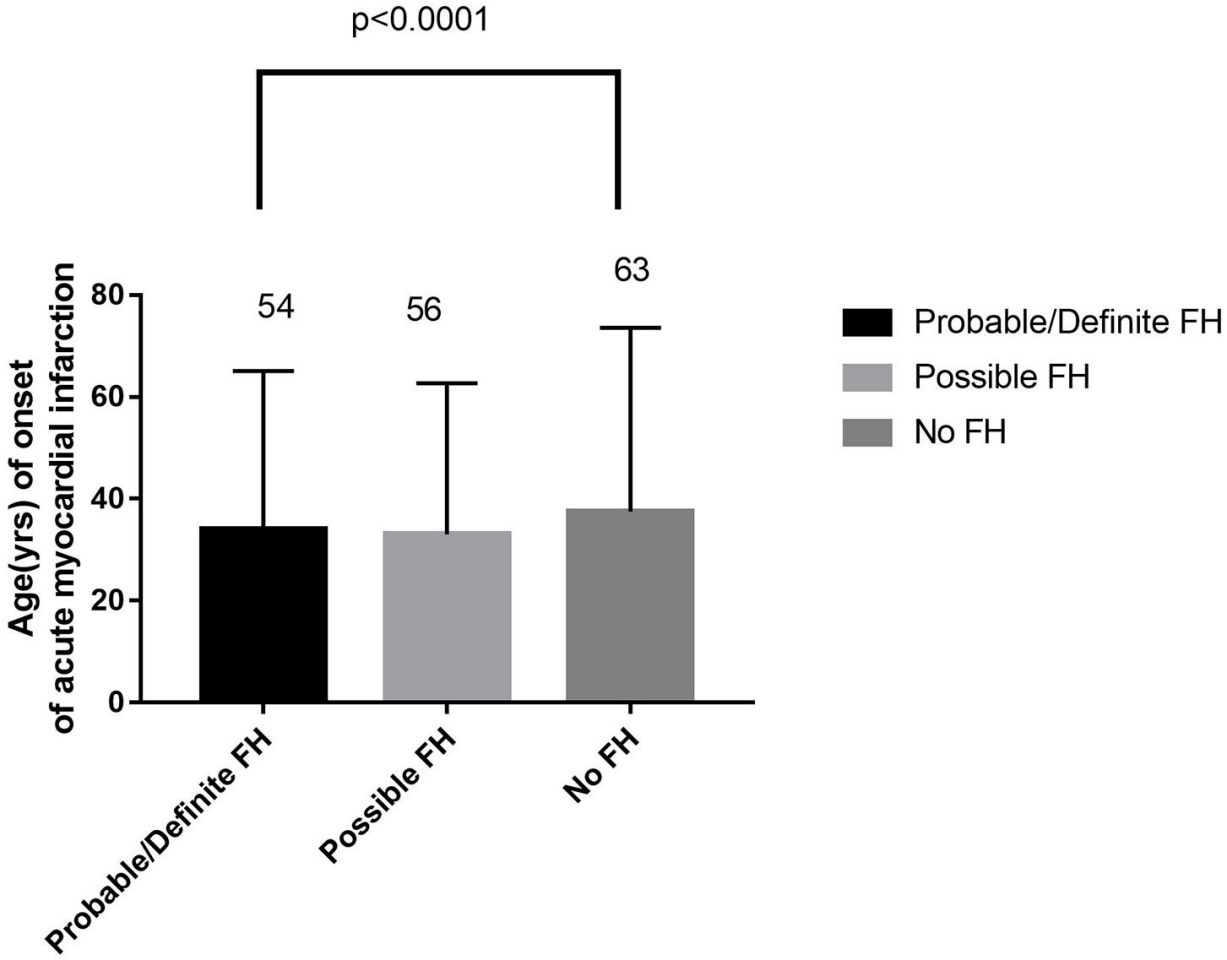
Comparison about age of onset of acute myocardial infarction in probable/definite HeFH, possible HeFH, and non-HeFH.

**Figure 4:**
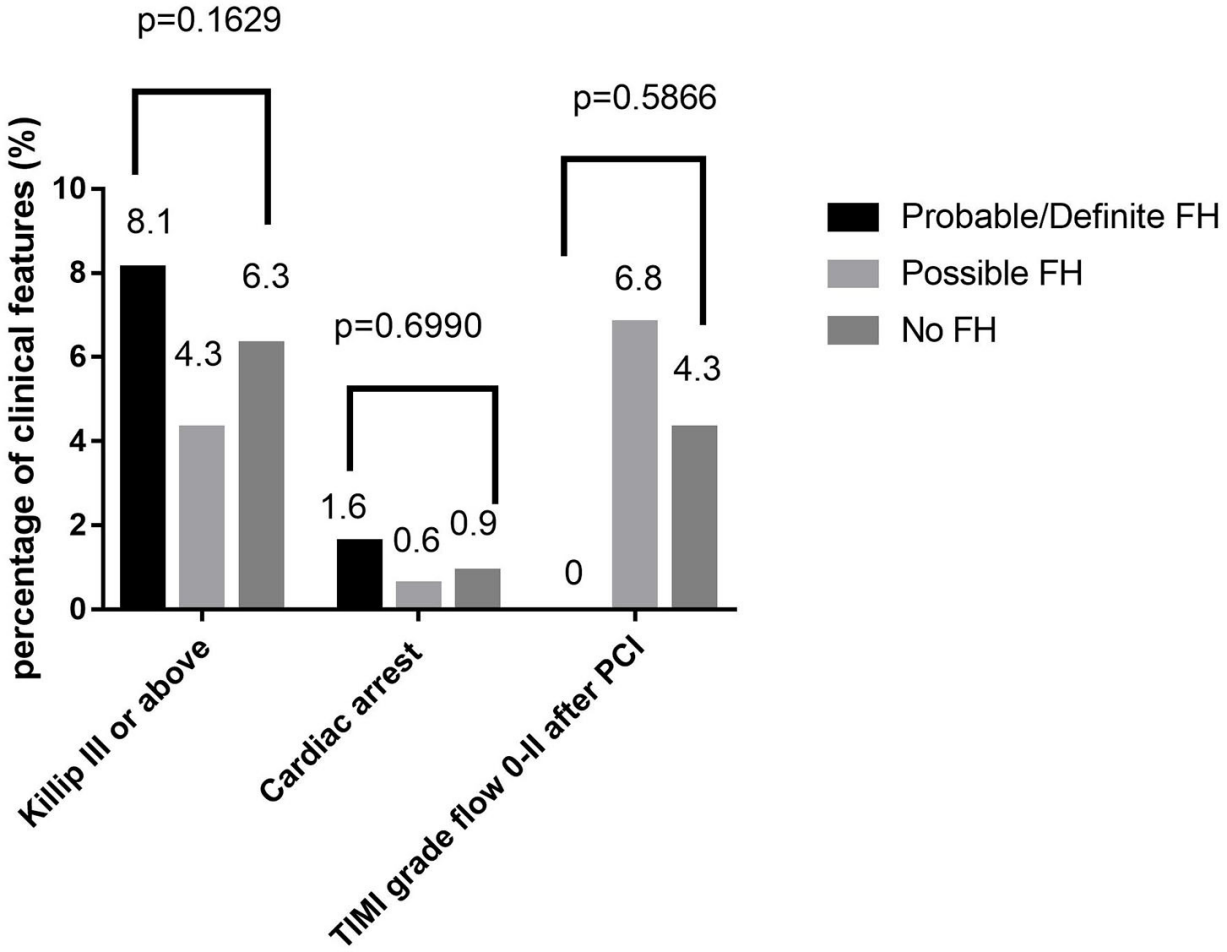
Comparison about severe clinical manifestations among probable/definite HeFH, possible HeFH, and non-HeFH groups.

The authors apologize for this error and state that this does not change the scientific conclusions of the article in any way. The original article has been updated.

